# Update of the activity of telavancin against a global collection of *Staphylococcus aureus* causing bacteremia, including endocarditis (2011–2014)

**DOI:** 10.1007/s10096-016-2865-8

**Published:** 2017-01-22

**Authors:** R. E. Mendes, H. S. Sader, J. I. Smart, M. Castanheira, R. K. Flamm

**Affiliations:** 1 JMI Laboratories, 345 Beaver Kreek Centre, Suite A, North Liberty, IA 52317 USA; 2 Theravance Biopharma US, Inc., South San Francisco, CA USA

## Abstract

The efficacy and safety of telavancin is under evaluation for the treatment of subjects with complicated *Staphylococcus aureus* bacteremia and *S. aureus* right-sided infective endocarditis. This study evaluated the telavancin activity against a global collection of *S. aureus* causing bloodstream infections (BSI), including endocarditis, to support the development of bacteremia/endocarditis clinical indications. This study included a total of 4191 *S. aureus* [1490 methicillin-resistant *S. aureus* (MRSA)], which were unique (one per patient) clinical isolates recovered from blood samples collected during 2011–2014 in a global network of hospitals. All isolates were deemed responsible for BSI, including endocarditis, by local guidelines. Isolates were tested for susceptibility by broth microdilution. Telavancin (MIC_50/90_, 0.03/0.06 μg/ml) inhibited all *S. aureus* at ≤0.12 μg/ml, the breakpoint for susceptibility. Equivalent minimum inhibitory concentration (MIC) values (MIC_50/90_, 0.03/0.06 μg/ml) were obtained for telavancin against methicillin-susceptible *S. aureus* (MSSA) and MRSA isolates, as well as MRSA from community and healthcare origins. Similar telavancin activities (MIC_50_, 0.03 μg/ml) were observed against MRSA subsets from North America and Europe, while isolates from the Asia-Pacific (APAC) and Latin America regions had MIC_50_ values of 0.06 μg/ml. MRSA with vancomycin MIC values of 2–4 μg/ml and the multidrug resistance (MDR) subset had telavancin MIC_50_ results of 0.06 μg/ml, although the MIC_100_ result obtained against these subsets remained identical to those of MSSA (MIC_100_, 0.12 μg/ml, respectively). This study updates the telavancin in vitro activity, which continues to demonstrate great potency against invasive *S. aureus*, regardless of the susceptibility phenotype or demographic characteristics (100.0% susceptible), and supports the sought-after subsequent indications.

## Introduction

*Staphylococcus aureus* is the second most common cause of bloodstream infection (BSI), and is the most important cause of BSI-associated death [1]. Prevalence-based studies, such as the SENTRY Antimicrobial Surveillance Program, observed a rate of 44.3% of *S. aureus* [45.4% methicillin-resistant *S. aureus* (MRSA)] causing bacteremia in USA hospitals during the 2015 sampling year (unpublished JMI data). Among multicenter and population-based investigations, incidence rates of 15–40 per 100,000 population per year have been identified, with case-fatality rates of approximately 15–25% [1–3].

Telavancin is a once-daily parenteral semi-synthetic lipoglycopeptide agent approved in the United States, Europe, and Canada for clinical indications, such as complicated skin and skin structure infections and/or hospital-acquired and ventilator-associated bacterial pneumonia (see package inserts for a complete description of respective indications) [4]. The efficacy and safety of telavancin is also under evaluation for the treatment of subjects with complicated *S. aureus* bacteremia and *S. aureus* right-sided infective endocarditis (NCT02208063) [5]. The in vitro activity of telavancin has been monitored and reported previously. However, this study evaluated the telavancin activity against a recent and global collection of *S. aureus* bacteremia isolates, including those responsible for endocarditis, to support the sought-after bacteremia/endocarditis clinical indications.

## Materials and methods

This study included a total of 4191 *S. aureus* (1490 MRSA), which were unique (one per patient) clinical isolates recovered from blood samples collected during 2011–2014 in a global network of hospitals in the North America (2150 isolates), Europe (1283), Latin America (473), and Asia-Pacific (APAC; 285) regions. All isolates were deemed responsible for BSI, including endocarditis, by the participating site according to local guidelines. Isolates that met the protocol selection criteria had the bacterial identification initially performed by the participating laboratory, which submitted isolates to a central monitoring laboratory (JMI Laboratories, North Liberty, IA, USA), as part of the SENTRY Antimicrobial Surveillance Program. Bacterial identification was subsequently confirmed by the reference monitoring laboratory by standard algorithms. Isolates showing questionable phenotypic and/or biochemical results had the bacterial identification confirmed by matrix-assisted laser desorption/ionization time-of-flight mass spectrometry (MALDI-TOF-MS; Bruker Daltonics, Bremen, Germany).

Isolates were tested for susceptibility by broth microdilution following the Clinical and Laboratory Standards Institute (CLSI) M07-A10 document [6]. Testing was performed using panels manufactured by Thermo Fisher Scientific (Cleveland, OH, USA). These validated panels provide minimum inhibitory concentration (MIC) results equivalent to the CLSI-approved broth microdilution method, which includes 0.002% polysorbate 80 in the testing media [6]. Bacterial inoculum density was monitored by colony counts to assure an adequate number of cells for each testing event. Quality of the MIC values was assured by concurrent testing of CLSI-recommended quality control (QC) reference strains (*S. aureus* ATCC 29213 and *Enterococcus faecalis* ATCC 29212) [6]. All QC results were within published acceptable ranges [6]. MIC interpretations for comparator agents were based on the CLSI M100-S26 [7] and European Committee on Antimicrobial Susceptibility Testing (EUCAST) [8] criteria, as available. Data analysis was performed by grouping isolates based on infection type, geographic region, and community-acquired (CA) and healthcare-associated (HA) origin based on the Centers for Disease Control and Prevention (CDC) criteria [9] and antimicrobial susceptibility phenotype. The latter applied the oxacillin breakpoint for grouping methicillin-susceptible (MSSA) and -resistant (MRSA) isolates and the vancomycin MIC results for segregating *S. aureus* between those with vancomycin MIC values of ≤1 or 2–4 μg/ml. Isolates were also categorized based on multidrug resistance (MDR), defined as MRSA isolates (methicillin [oxacillin]-resistant) resistant to an additional three or more drug classes (see Table 2 for a complete list of antimicrobials utilized).

## Results and discussion

Overall, *S. aureus* isolates were 100.0% susceptible to telavancin and had the highest MIC_50_, MIC_90_, and MIC_100_ results of 0.03, 0.06, and 0.12 μg/ml, respectively. Equivalent MIC values (MIC_50/90_, 0.03/0.06 μg/ml) were obtained for telavancin against isolates from different infection types (i.e., BSI and endocarditis), MSSA and MRSA isolates, as well as MRSA from CA and HA origins (Table 1). Telavancin (MIC_50/90_, 0.03/0.06 μg/ml) had similar potency against all *S. aureus* and the MRSA subsets from North America and Europe. The isolates from the APAC and Latin America regions had slightly higher MIC_50_ values (MIC_50/90_, 0.06/0.06 μg/ml), although the MIC_90_ and MIC_100_ results remained identical to those obtained for the overall MSSA population (Table 1).

**Table 1:** Antimicrobial activity and minimum inhibitory concentration (MIC) distributions for telavancin when tested against *Staphylococcus aureus* clinical isolates, as part of the international telavancin surveillance program

*S. aureus*/Parameter (number tested)	MIC (μg/ml)		Number (cumulative %) inhibited at a telavancin MIC (μg/ml) of:			
	50%	90%	≤0.015	0.03	0.06	0.12
All (4191)	0.03	0.06	169 (4.0)	2465 (62.8)	1538 (99.5)	19 (100.0)
Infection type						
BSI (4149)	0.03	0.06	166 (4.0)	2444 (62.9)	1520 (99.5)	19 (100.0)
Endocarditis (42)	0.03	0.06	3 (7.1)	21 (57.1)	18 (100.0)	
Origin						
CA-MRSA (828)	0.03	0.06	24 (2.9)	482 (61.1)	312 (98.8)	10 (100.0)
HA-MRSA (552)	0.03	0.06	15 (2.7)	284 (54.2)	250 (99.5)	3 (100.0)
Phenotype						
MSSA (2701)	0.03	0.06	127 (4.7)	1632 (65.1)	938 (99.9)	4 (100.0)
MRSA (1490)	0.03	0.06	42 (2.8)	833 (58.7)	600 (99.0)	15 (100.0)
MDR (569)	0.06	0.06	8 (1.4)	264 (47.8)	285 (97.9)	12 (100.0)
Non-MDR (921)	0.03	0.06	34 (3.7)	569 (65.5)	315 (99.7)	3 (100.0)
Vancomycin MIC ≤1 μg/ml (1439)	0.03	0.06	41 (2.8)	828 (60.4)	561 (99.4)	9 (100.0)
Vancomycin MIC = 2–4 μg/ml (51)	0.06	0.12	1 (2.0)	5 (11.8)	39 (88.2)	6 (100.0)
Region						
North America (2150)	0.03	0.06	102 (4.7)	1374 (68.7)	662 (99.4)	12 (100.0)
MRSA (938)	0.03	0.06	30 (3.2)	586 (65.7)	311 (98.8)	11 (100.0)
Europe (1283)	0.03	0.06	52 (4.1)	798 (66.3)	430 (99.8)	3 (100.0)
MRSA (290)	0.03	0.06	10 (3.4)	174 (63.4)	105 (99.7)	1 (100.0)
Latin America (473)	0.06	0.06	13 (2.7)	206 (46.3)	250 (99.2)	4 (100.0)
MRSA (175)	0.06	0.06	1 (0.6)	47 (27.4)	124 (98.3)	3 (100.0)
APAC (285)	0.06	0.06	2 (0.7)	87 (31.2)	196 (100.0)	
MRSA (87)	0.06	0.06	1 (1.1)	26 (31.0)	60 (100.0)	

*BSI* bloodstream infection, *MSSA* methicillin-susceptible *S. aureus*, *MRSA* methicillin-resistant *S. aureus*, *CA-MRSA* community-acquired MRSA, *HA-MRSA* healthcare-associated MRSA; the origin of the isolate was defined based on CDC criteria, *MDR* multidrug resistance, defined as MRSA (methicillin [oxacillin]-resistant) resistant to three or more drug classes in addition to β-lactam agents

MRSA with vancomycin MIC values of 2–4 μg/ml and the MDR subset had telavancin MIC_50_ results of 0.06 μg/ml, which was 2-fold higher than the telavancin MIC_50_ results (MIC_50_, 0.03 μg/ml) for the isolates exhibiting vancomycin MIC values at ≤1 μg/ml or a non-MDR phenotype. Even though the MIC_50_ was higher in these resistant subgroups, telavancin still inhibited all isolates at the susceptible breakpoint of ≤0.12 μg/ml (Table 1). Daptomycin (MIC_50/90_, 0.5/1 μg/ml; Table 2) also demonstrated higher MIC results when tested against MRSA exhibiting vancomycin MIC values of 2–4 μg/ml compared with those isolates with vancomycin MIC values of ≤1 μg/ml (data not shown).

**Table 2:** Antimicrobial activity of telavancin and comparator agents tested against a global collection of resistant subsets of *S. aureus* clinical isolates responsible for bloodstream infections, including endocarditis

Organism (number tested)/antimicrobial agent	MIC (μg/ml)			% susceptible/% intermediate/% resistant^a^					
	Range	50%	90%	CLSI			EUCAST		
MRSA (1490)									
Telavancin	≤0.015–0.12	0.03	0.06	100.0	–	–^b^	100.0	–	0.0
Clindamycin	≤0.25–>2	≤0.25	>2	63.7	0.2	36.1	63.5	0.2	36.3
Daptomycin	0.12–2	0.25	0.5	99.7	–	–	99.7	–	0.3
Erythromycin	≤0.12–>16	>16	>16	18.8	2.8	78.4	19.1	0.6	80.3
Gentamicin	≤1–>8	≤1	>8	87.7	0.4	11.9	87.3	–	12.7
Levofloxacin	≤0.12–>4	>4	>4	24.9	1.5	73.6	24.9	1.5	73.6
Linezolid	0.25–4	1	1	100.0	–	0.0	100.0	–	0.0
Tetracycline	≤0.5–>8	≤0.5	2	92.3	0.4	7.3	89.6	2.2	8.1
TMP-SMX	≤0.5–>4	≤0.5	≤0.5	97.7	–	2.3	97.7	0.1	2.1
Vancomycin	0.25–4	1	1	99.9	0.1	0.0	99.9	–	0.1
MRSA MDR (569)									
Telavancin	≤0.015–0.12	0.06	0.06	100.0	–	–	100.0	–	0.0
Clindamycin	≤0.25–>2	>2	>2	9.3	0.4	90.3	9.0	0.4	90.7
Daptomycin	0.12–2	0.25	0.5	99.3	–	–	99.3	–	0.7
Erythromycin	0.5–>16	>16	>16	0.5	1.6	97.9	0.5	0.5	98.9
Gentamicin	≤1–>8	≤1	>8	71.9	0.5	27.6	71.5	–	28.5
Levofloxacin	≤0.12–>4	>4	>4	0.9	0.5	98.6	0.9	0.5	98.6
Linezolid	0.25–4	1	1	100.0	–	0.0	100.0	–	0.0
Tetracycline	≤0.5–>8	≤0.5	>8	89.1	0.0	10.9	84.0	5.1	10.9
TMP-SMX	≤0.5–>4	≤0.5	≤0.5	94.6	–	5.4	94.6	0.4	5.1
Vancomycin	0.5–4	1	1	99.8	0.2	0.0	99.8	–	0.2
MRSA with vancomycin MIC = 2–4 μg/ml (51)									
Telavancin	≤0.015–0.12	0.06	0.12	100.0	–	–	100.0	–	0.0
Clindamycin	≤0.25–>2	>2	>2	25.5	0.0	74.5	25.5	0.0	74.5
Daptomycin	0.25–2	0.5	1	98.0	–	–	98.0	–	2.0
Erythromycin	≤0.12–>16	>16	>16	9.8	2.0	88.2	9.8	2.0	88.2
Gentamicin	≤1–>8	≤1	>8	72.5	0.0	27.5	72.5	–	27.5
Levofloxacin	≤0.12–>4	>4	>4	13.7	0.0	86.3	13.7	0.0	86.3
Linezolid	0.25–2	1	2	100.0	–	0.0	100.0	–	0.0
Tetracycline	≤0.5–>8	≤0.5	2	90.2	0.0	9.8	84.3	5.9	9.8
TMP-SMX	≤0.5–>4	≤0.5	≤0.5	94.1	–	5.9	94.1	0.0	5.9
Vancomycin	2–4	2	2	98.0	2.0	0.0	98.0	–	2.0

*MRSA* methicillin-resistant *S. aureus*, *TMP-SMX* trimethoprim–sulfamethoxazole, *MDR* multidrug resistance (defined as MRSA resistant to three or more drug classes in addition to β-lactam agents)

^a^Breakpoint criteria according to the CLSI (M100-S26, 2016) and EUCAST, as available

**Fig. 1:**
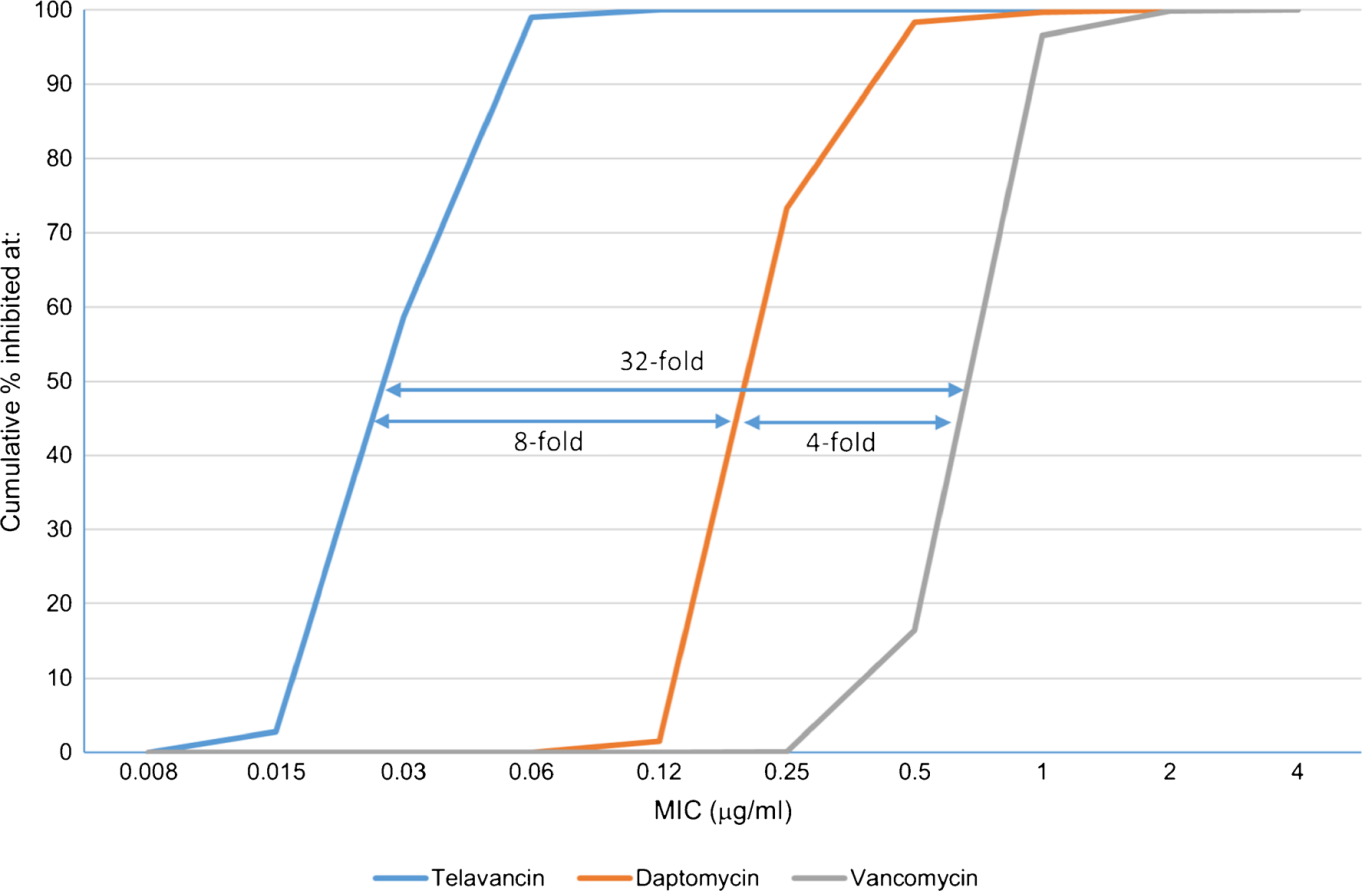
Telavancin, daptomycin, and vancomycin minimum inhibitory concentration (MIC) distributions obtained against all bacteremia methicillin-resistant *Staphylococcus aureus* (MRSA). Data are presented as the cumulative percentage of isolates inhibited at each MIC (μg/ml). MIC_50_ differences between drugs are depicted

Overall, telavancin showed MIC_50_ results 8-fold lower than daptomycin (MIC_50/90_, 0.25/0.5 μg/ml) and up to 32-fold lower than vancomycin (MIC_50/90_, 1/1 μg/ml) against bacteremia MRSA, including those causing endocarditis (Fig. 1 and Table 2). Similarly, the telavancin MIC results (MIC_50/90_, 0.06/0.06 μg/ml) were 4- to 8-fold lower than those obtained by daptomycin (MIC_50/90_, 0.25/0.5 μg/ml) and 16-fold lower than vancomycin (MIC_50/90_, 1/1 μg/ml) against the MRSA MDR subset (Table 2). When tested against the MRSA subset displaying decreased susceptibility to vancomycin (MIC, 2–4 μg/ml), telavancin (MIC_50/90_, 0.06/0.12 μg/ml) and daptomycin (MIC_50/90_, 0.5/1 μg/ml) were the most potent agents; however, telavancin was 8-fold more potent than daptomycin (Table 2).

Telavancin demonstrated potent in vitro activity against this contemporary global collection of *S. aureus* causing bacteremia, including resistant subsets and isolates causing endocarditis. In addition, telavancin had in vitro potency at least 4-fold greater than other clinically available comparator antimicrobial agents (daptomycin and vancomycin) recommended by the current Infectious Diseases Society of America (IDSA) guidelines for the treatment of bacteremia caused by MRSA and MDR subsets [10]. These in vitro results support further investigations of telavancin as a candidate for the treatment of bacteremia caused by *S. aureus* and resistant subsets, including those isolates responsible for endocarditis [11]. Moreover, the results obtained for telavancin corroborate those reported previously and indicate sustained in vitro potency over time against *S. aureus* isolates causing infections in hospitals worldwide [12–14].

## References

[1] Tong SY, Davis JS, Eichenberger E, Holland TL, Fowler VG (2015). *Staphylococcus aureus* infections: epidemiology, pathophysiology, clinical manifestations, and management. Clin Microbiol Rev.

[2] Laupland KB, Lyytikäinen O, Søgaard M, Kennedy KJ, Knudsen JD, Ostergaard C, Galbraith JC, Valiquette L, Jacobsson G, Collignon P, Schønheyder HC, International Bacteremia Surveillance Collaborative (2013). The changing epidemiology of *Staphylococcus aureus* bloodstream infection: a multinational population-based surveillance study. Clin Microbiol Infect.

[3] Klevens RM, Morrison MA, Nadle J, Petit S, Gershman K, Ray S, Harrison LH, Lynfield R, Dumyati G, Townes JM, Craig AS, Zell ER, Fosheim GE, McDougal LK, Carey RB, Fridkin SK, Active Bacterial Core surveillance (ABCs) MRSA Investigators (2007). Invasive methicillin-resistant *Staphylococcus aureus* infections in the United States. JAMA.

[4] VIBATIV Package Insert (2016) Theravance

[5] Friedman B, Bressler A, Cleveland K, Lat A, Helgeson M, Sherman C, Castaneda-Ruiz B (2016). 686: telavancin observational use registry: preliminary results for bacteremia and endocarditis. Crit Care Med.

[6] Clinical and Laboratory Standards Institute (CLSI) (2015). M07-A10. Methods for dilution antimicrobial susceptibility tests for bacteria that grow aerobically; approved standard—tenth edition.

[7] Clinical and Laboratory Standards Institute (CLSI) (2015). M100-S25. Performance standards for antimicrobial susceptibility testing; twenty-fifth informational supplement.

[8] European Committee on Antimicrobial Susceptibility Testing (EUCAST) (2016) Breakpoint tables for interpretation of MICs and zone diameters. Version 6.0, January 2016. Available online at: http://www.eucast.org/clinical_breakpoints/. Accessed Jan 2016

[9] Kreisel K, Roghmann MC, Shardell M, Stine OC, Perencevich E, Lesse A, Gordin F, Climo M, Johnson JK (2010). Assessment of the 48-hour rule for identifying community-associated methicillin-resistant *Staphylococcus aureus* infection complicated by bacteremia. Infect Control Hosp Epidemiol.

[10] Liu C, Bayer A, Cosgrove SE, Daum RS, Fridkin SK, Gorwitz RJ, Kaplan SL, Karchmer AW, Levine DP, Murray BE, J Rybak M, Talan DA, Chambers HF, Infectious Diseases Society of America (2011). Clinical practice guidelines by the Infectious Diseases Society of America for the treatment of methicillin-resistant *Staphylococcus aureus* infections in adults and children. Clin Infect Dis.

[11] Corey GR, Rubinstein E, Stryjewski ME, Bassetti M, Barriere SL (2015). Potential role for telavancin in bacteremic infections due to gram-positive pathogens: focus on *Staphylococcus aureus*. Clin Infect Dis.

[12] Mendes RE, Sader HS, Flamm RK, Farrell DJ, Jones RN (2015). Telavancin in vitro activity against a collection of methicillin-resistant *Staphylococcus aureus* isolates, including resistant subsets, from the United States. Antimicrob Agents Chemother.

[13] Mendes RE, Farrell DJ, Sader HS, Streit JM, Jones RN (2015). Update of the telavancin activity in vitro tested against a worldwide collection of Gram-positive clinical isolates (2013), when applying the revised susceptibility testing method. Diagn Microbiol Infect Dis.

[14] Mendes RE, Flamm RK, Farrell DJ, Sader HS, Jones RN (2016). Telavancin activity tested against Gram-positive clinical isolates from European, Russian and Israeli hospitals (2011–2013) using a revised broth microdilution testing method: redefining the baseline activity of telavancin. J Chemother.

